# The origin of the double-muscle mutation in the *MSTN* gene of sheep and its effects on growth traits in the Hu × East Friesian hybrid population

**DOI:** 10.3389/fvets.2026.1791780

**Published:** 2026-04-29

**Authors:** Yuan Xu, Caiyue Gao, Haotian Wang, Chunna Cao, Xinyue Zhang, Baohang Sun, Yilin Liang, Yuheng Bai, Yuhe Li, Yu Jiang, Ran Li

**Affiliations:** 1 Key Laboratory of Animal Genetics, Breeding and Reproduction of Shaanxi Province, College of Animal Science and Technology, Northwest A&F University, Yangling, Shaanxi, China; 2 College of Animal Engineering, Shaanxi A&F Technology University, Key Laboratory for Efficient Ruminant Breeding Technology of Higher Education Institutions in Shaanxi Province, Yangling, Shaanxi, China; 3 Yulin Sheep Industry Development Center, Yulin, Shaanxi, China

**Keywords:** ancestral recombination graph, double-muscle, evolutionary origin, growth traits, sheep genomics

## Abstract

**Introduction:**

The g.119292288G > A mutation in the 3′ untranslated region (3′UTR) of the myostatin (*MSTN*) gene is a well-known functional variant associated with increased muscling in Texel sheep. However, its evolutionary origin and historical dynamics remain unresolved, which are critical for understanding the genomic architecture of major-effect mutations and their implications for animal breeding.

**Methods:**

We analyzed whole-genome sequencing data from 73 diverse global sheep breeds to characterize the allele frequency distribution of the *MSTN* g.119292288G>A mutation. Selection history was inferred using ancestral recombination graph–based approaches implemented in Relate and CLUES. To assess the temporal origin of the mutation, we screened 31 ancient European sheep genomes dated between 8,200 and 440 years before present. The phenotypic effects of the mutation were evaluated using association analyses in a Hu × East Friesian hybrid population.

**Results:**

The derived allele (A) showed a high frequency (>0.7) in East Friesian, Texel, and Dairy Meade sheep, but was rare or absent in other breeds, suggesting a likely origin in the northern Netherlands, particularly in the regions of Texel Island and Friesland Province. Selection analyses indicated that this allele experienced strong positive selection within the last ~300 years. Notably, neither the mutation nor its associated haplotype was detected in any of the ancient European samples, supporting a relatively recent emergence. In the Hu × East Friesian hybrid population, individuals carrying the mutant allele exhibited significantly greater birth weight, weaning weight, and six-month body weight compared to wild-type homozygotes (*p* < 0.05).

**Conclusion:**

Together, our results provide a comprehensive overview of the origin, spread, and phenotypic impact of the *MSTN* g.119292288G > A mutation, offering valuable insights for identifying and utilizing major-effect mutations in genomic breeding programs.

## 1 Introduction

The *MSTN* gene, a member of the transforming growth factor-*β* (TGF-β) superfamily (1) and also referred to as growth differentiation factor 8 (*GDF8*), is a well-established negative regulator of skeletal muscle growth (2) and plays a critical role in shaping muscle-related traits across species (3, 4). Mutations in the *MSTN* gene have been found to have profound implications for muscle growth and development in various species (5). Mutations in *MSTN* can lead to a reduction or complete loss of the biological activity of myostatin (6), which in turn, allows for unregulated proliferation and differentiation of muscle cells, ultimately promoting muscle growth and often resulting in a double-muscle phenotype (4). While this double-muscling trait typically stems from coding region mutations impairing gene function, such as the 11-nucleotide deletion in *MSTN* exon 3 of Belgian Blue cattle that causes frameshift mutation, myostatin dysfunction, and double-muscling (7), the regulatory region (e.g., 3′UTR of *MSTN* in Texel sheep) can also induce this phenotype (8). The similar muscle hypertrophy phenotype has been observed in cattle (9), sheep (10), dogs (11), and pigs (12), which exhibit increased lean meat yield, higher muscle density, and lower fat content compared to their conventional counterparts. Transgenic sheep with significantly increased meat production have been successfully cultivated by knocking out the *MSTN* gene (13).

In Texel sheep, a point mutation in the 3′UTR of *MSTN* (g. + 6723G > A) creates a novel binding site for miR-1 and miR-206, leading to translational repression of *MSTN* and contributing to the characteristic muscular hypertrophy of the breed (8). Understanding the evolutionary origin and selection trajectory of functionally important mutations is crucial for identifying targets of past adaptation and for informing future trait improvement strategies. In human populations, studies focusing on major-effect mutations such as the lactase persistence allele (−13.910*T) (14), the rs174546 variant in *FADS1* affecting fatty acid metabolism (15), and the CCR5delta32 deletion conferring HIV resistance (16), have illustrated how tracing the emergence and spread of these mutations can illuminate responses to environmental and cultural pressures. In contrast, the selection history of similar mutations in livestock remains poorly understood. By tracing the origin and evolutionary history of the *MSTN* g.119292288G > A mutation across diverse sheep populations through phylogenetic and selection analyses, we aimed to assess its evolutionary conservation and evaluate its potential role as a target of artificial selection for muscle-related traits. This will help reveal the patterns of major-effect mutations in domestic animals and provide insights for the discovery and application of such important variants.

In this study, we performed an analysis of the frequency of this mutation across global sheep breeds. We also carried out population structure, selection signal, and haplotype analysis on European sheep. Moreover, by applying ARG-based dating methods to a globally representative sheep genomic dataset, we reconstructed the spatiotemporal dynamics of the *MSTN* mutation, tracing its emergence and spread over time. We strove to uncover the correlation between the *MSTN* g.119292288G > A mutation and the growth traits of the Hu × East Friesian hybrid population, providing a scientific basis for improving sheep meat quality and enhancing production efficiency.

## 2 Materials and methods

### 2.1 Modern genomic data collection and processing

Modern genomic data from 2,946 individuals representing 73 sheep breeds worldwide were obtained from the NCBI database. The fastp v0.24.0 (17) software was used to remove adapters, and the data were aligned to the sheep reference genome ARS-UI_Ramb_v2.0 using BWA-MEM v0.7.5a (18) software. BAM files were sorted using SAMtools v1.18 (19) software. Picard v2.21.2 (https://broadinstitute.github.io/picard/) was applied to mark and discard duplicate reads. Single nucleotide polymorphisms (SNPs) were called via the HaplotypeCaller module in GATK v4.1.4.1 (20) software. Raw gVCF files were merged with CombineGVCFs and joint genotyping was performed using GenotypeGVCFs. SNPs hard filtering was performed with GATK v4.1.4.1 (20) software using the criterion ‘QD < 2.0 || FS > 60.0 || MQ < 40.0 || MQRankSum < −12.5 || ReadPosRankSum < −8.0 || SOR > 3.0’. Using BCFtools v1.17 (19), biallelic sites with minor allele frequency (MAF) > 0.01 and a missing rate of less than 0.1 were extracted, resulting in the VCF file for subsequent analysis. We annotated the filtered SNPs using ANNOVAR (21) software to determine their genomic locations and potential functional effects.

### 2.2 Allele frequency analysis of the *MSTN* g.119292288G > A mutation

Using VCFtools v0.1.13 (22), we analyzed whole-genome sequencing data from modern sheep to calculate the allele frequency distribution of the *MSTN* g.119292288G > A mutation across 73 different sheep breeds worldwide.

### 2.3 Population genetic structure analysis in European sheep

A total of 168 European sheep were selected for population structure analysis, including Australian Merino sheep (13), Baikal Fine Fleeced sheep (20), East Friesian sheep (19), Finn sheep (14), German Merino sheep (13), Ouessant sheep (12), Poll Dorset sheep (19), Romney sheep (20), Suffolk sheep (14), and Texel sheep (19). A total of 26,556,340 SNPs were used for the analysis. Population genetic structure based on filtering to obtain high-quality SNPs was performed using Plink v1.90 (23) software to prune linkage disequilibrium (LD), with the parameter “--indep-pairwise 50 5 0.2”. After LD pruning, 2,584,991 SNPs remained. The smartPCA program in the EIGENSOFT v5.0 (24) package was used to perform principal component analysis (PCA). The maximum likelihood (ML) phylogenetic tree was inferred via IQ-TREE v2.2.2.7 (25), with the best-fit substitution model selected by ModelFinder based on SNP alignment data. Finally, the iTOL (26) website was used to visualize the tree. ADMIXTURE v1.3.0 (27) software was used to infer the population genetic structure of European sheep. The genetic structure was calculated for K values ranging from 2 to 9. We selected the K value with the lowest cross-validation (CV) error as the optimal number of ancestral populations in the ADMIXTURE analysis.

### 2.4 Selection signal and haplotype analysis between Texel, East Friesian, and other European sheep breeds

To calculate the genome-wide sliding window *F*_ST_ between Texel, East Friesian, and other European sheep breeds using VCFtools v0.1.13 (22), with a window size of 10 kb and a step size of 2 kb. The top 1% of weighted *F*_ST_ values were identified as candidate regions under strong selection. We used Selscan v2.0.2 (28) to calculate the global XPEHH values. We selected the windows with absolute XPEHH values > 1 as the regions under selection in Texel and East Friesian sheep. The overlapping regions under selection identified by the two selection methods were annotated using the ANNOVAR (21) software. Finally, using the Asiatic mouflon as a control population, a python script was employed to visualize the haplotypes of the linkage regions in other European sheep breeds.

### 2.5 Mutation age estimation of the *MSTN* g.119292288G > A mutation

For this SNP, we first used Relate v1.19 (29) software to extract the corresponding local tree and resampled branch lengths. The analysis was performed assuming an effective population size (Ne) of 5,000 and a mutation rate of 1.51 × 10^−8^ per site per generation. Branch length resampling was conducted using a painting parameter of 0.025 1, and a fixed random seed of 1 was used to ensure reproducibility. CLUES (30) software calculates a likelihood ratio for the SNP, reflecting the degree to which the derived allele of this SNP deviates from the neutral mutation model. The resampled genealogies and the corresponding.coal file were provided as input. Time discretization was specified using predefined time bins (--timeBins), and trajectories were inferred up to a cutoff time of 2,000 generations (--tCutoff 2000). The smc++ v1.15.5 (31) software was utilized to calculate the divergence time between the breeds containing this mutation and those without it, aiming to understand the time of differentiation among different breeds. The mutation rate was 1.51 × 10^−8^, with a generation time of three years (32).

### 2.6 Ancient European genomic data processing

Single reads were trimmed with Cutadapt v4.6 (33), and paired-end reads were trimmed with AdapterRemoval v2.3.3 (34). Clean reads were aligned to the ARS-UI_Ramb_v2.0 genome using BWA-MEM v0.7.5a (18). PCR duplicates were removed with Picard v2.21.2, and indel realignment was performed using GATK v4.1.4.1 (20). Reads with mapping quality below 30 were filtered using SAMtools v1.18 (19). Autosomal coverage was calculated with the DepthOfCoverage module from GATK v4.1.4.1 (20). Genotype calling for ancient samples was performed using BCFtools v1.17 (19), applying the mpileup and call functions to these predefined SNP positions. Following variant calling, only biallelic SNPs were retained for subsequent analyses. We used GLIMPSE2 (35) to impute the collected ancient European samples based on an internal sheep reference panel. To investigate the temporal dynamics of the selected regions, we visualized the haplotype structure using genotypes from ancient individuals, with samples sorted by age.

### 2.7 Collection of phenotypic data and correlation analysis between the *MSTN* g.119292288G > A mutation and growth traits

All animal procedures were conducted in accordance with the guidelines of the Administration of Affairs Concerning Experimental Animals of China and were approved by the Institutional Animal Care and Use Committee of Northwest A&F University (IACUC-NWAFU).

Phenotypic data obtained from Shaanxi Shanghe Sannong Science and Technology Co., Ltd. underwent rigorous quality control, including the removal of missing values, erroneous entries, and outliers, to ensure data integrity and analytical accuracy. A total of 853 male Hu × East Friesian hybrid individuals were evaluated for ten growth traits. These traits included body height, body length, chest depth, chest width, chest circumference, and cannon bone circumference—measured at two months of age—as well as birth weight, weaning weight, average daily gain before weaning, and six-month body weight. All experimental individuals were derived from a single Hu × East Friesian hybrid population and were raised under identical feeding and management conditions. The mutation was genotyped using Kompetitive Allele Specific PCR (KASP) technology by COMPASS BIOTECHNOLOGY Co., Ltd.

A linear model was employed in SPSS v27.0 to analyze the association between the genotype at the *MSTN* g.119292288G > A mutation and growth traits. The results were expressed as the means ± standard errors, with *p* < 0.05 used as the criterion for significance. The formula is as follows: $Y_{\text{ijlk}}=\mu +A_{i}+B_{j}+M_{l}+G_{k}+e_{\mathrm{ijk}}$ . In the equation, Y_ijlk_ represents the individual phenotypic record; *μ* is the mean of each trait in the population; A_i_ and B_j_ denote the effects of litter size and sex; M_l_ denotes the effect of birth year and month; G_k_ is the genotype effect; and e_ijk_ is the random error.

## 3 Results

### 3.1 Frequency distribution of the *MSTN* g.119292288G > A mutation

**Figure 1:**
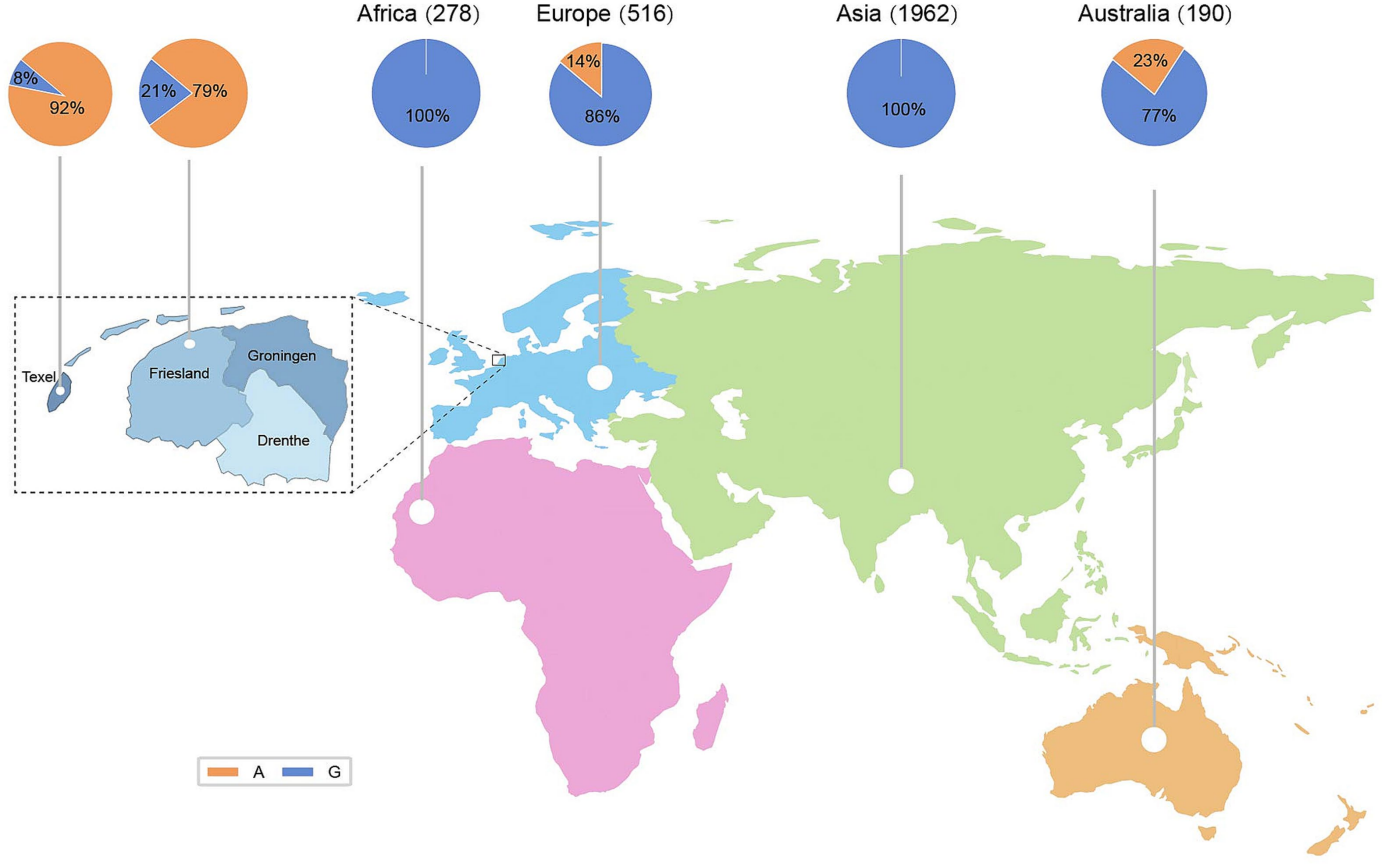
Geographic distribution frequency of the *MSTN* g.119292288G > A mutation.

Based on the genomic data from 73 sheep breeds worldwide, we generated a frequency distribution table and diagram for the *MSTN* g.119292288G > A mutation (Supplementary Table 1) (Figure 1). The mutation frequency was primarily observed in European (0.86) and Australian (0.77) populations but was nearly absent in Asian populations (Figure 1). It was particularly prevalent in two northern European breeds—Texel (0.91) and East Friesian (0.79)—as well as in Dairy Meade sheep (0.84), a composite breed developed through crossbreeding East Friesian sheep with other breeds (Supplementary Table 1). The A allele was predominant in East Friesian (*n* = 56), Texel (*n* = 29), and Dairy Meade sheep (*n* = 31). In contrast, the mutation was present at a low frequency in Australian White (*n* = 119) and Coopworth sheep (*n* = 40). Among the remaining 2,671 individuals sampled from other global breeds, the mutation was either rare or entirely absent (Supplementary Table 1). The highest mutation frequency was detected in Texel and East Friesian sheep, both of which originated from the northern Netherlands, particularly Texel Island and Friesland Province. The high prevalence of this mutation in these two geographically proximate breeds suggested that it most likely originated in this region.

### 3.2 Population structure of European sheep

**Figure 2:**
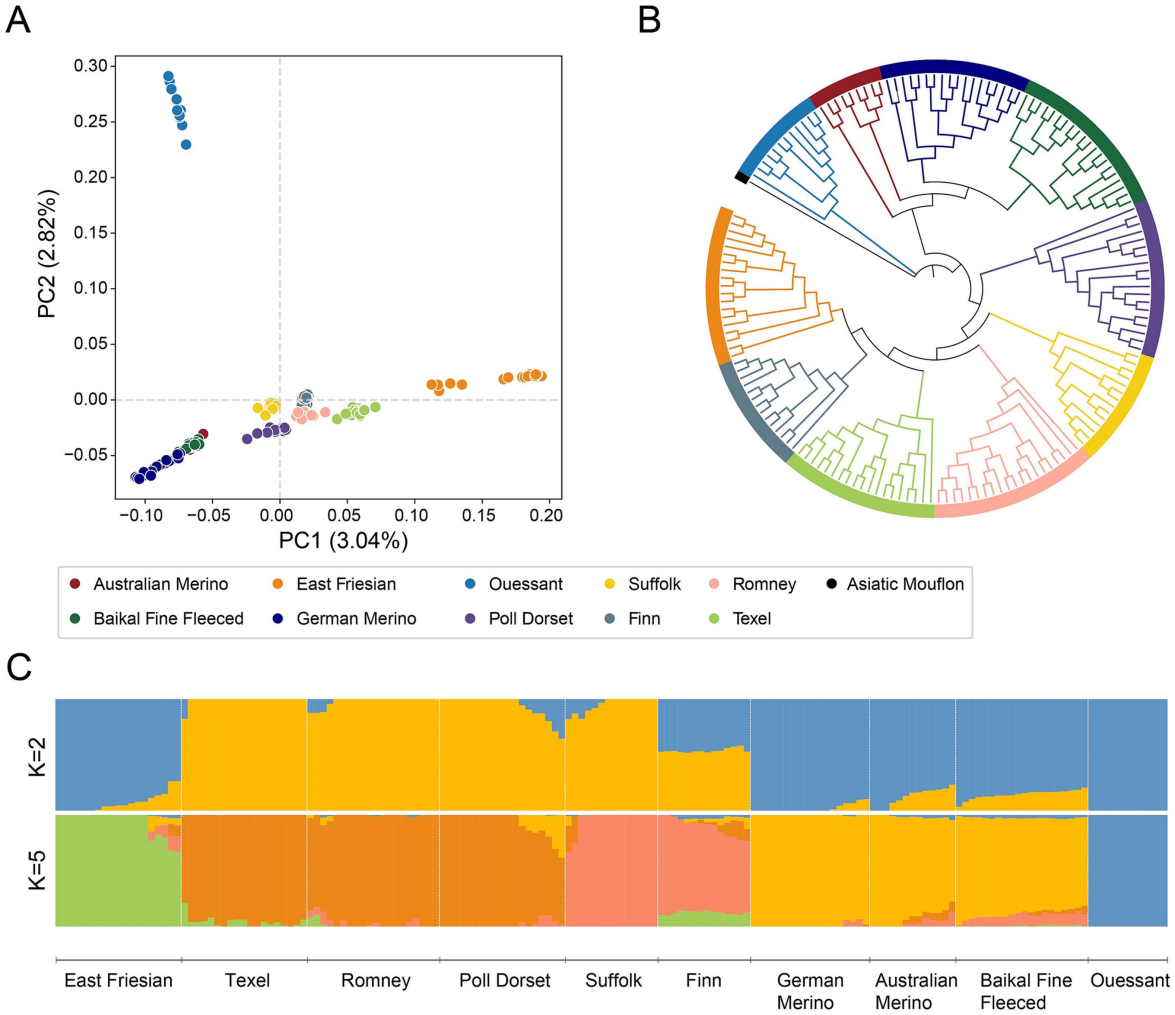
Population structure of European sheep **(A)** PCA of European sheep; **(B)** Maximum likelihood tree of European sheep breeds; **(C)** ADMIXTURE results for K = 2 and K = 5, showing low cross-validation error.

Given the high prevalence of the *MSTN* g.119292288G > A mutation in specific European breeds, we next investigated the population structure of these and other related breeds to better understand the genetic background and potential origin of the mutation. PCA revealed three distinct clusters (Figure 2A). Based on the first principal component, PC1 (3.04%), European sheep primarily clustered into wool breeds with Merino ancestry and meat breeds represented by Texel sheep. Notably, Texel and East Friesian sheep were located on the same side of PC1, indicating a degree of genetic similarity. For the second principal component, PC2 (2.82%), Ouessant formed a separate cluster.

The maximum likelihood (ML) tree further confirmed this global population structure (Figure 2B). Merino sheep and its derivatives (Baikal Fine Fleeced, German Merino and Australian Merino) grouped together, reflecting their shared ancestry. Texel sheep clustered with other meat breeds, such as Romney and Poll Dorset sheep, whereas East Friesian sheep appeared closely related to Texel sheep, despite their dairy background. Ouessant sheep formed a distinct branch, consistent with previous studies showing its genetic divergence (36).

In the ADMIXTURE analysis (Figure 2C), when K = 2, European sheep were divided into two major groups: meat breeds and wool breeds. When K = 5, East Friesian sheep were clearly distinct from other European sheep, exhibiting unique ancestral components. This suggested that, as a dairy breed, it might have undergone intensive artificial selection during the later stages.

### 3.3 Shared selective sweeps in Texel and East Friesian sheep

**Figure 3:**
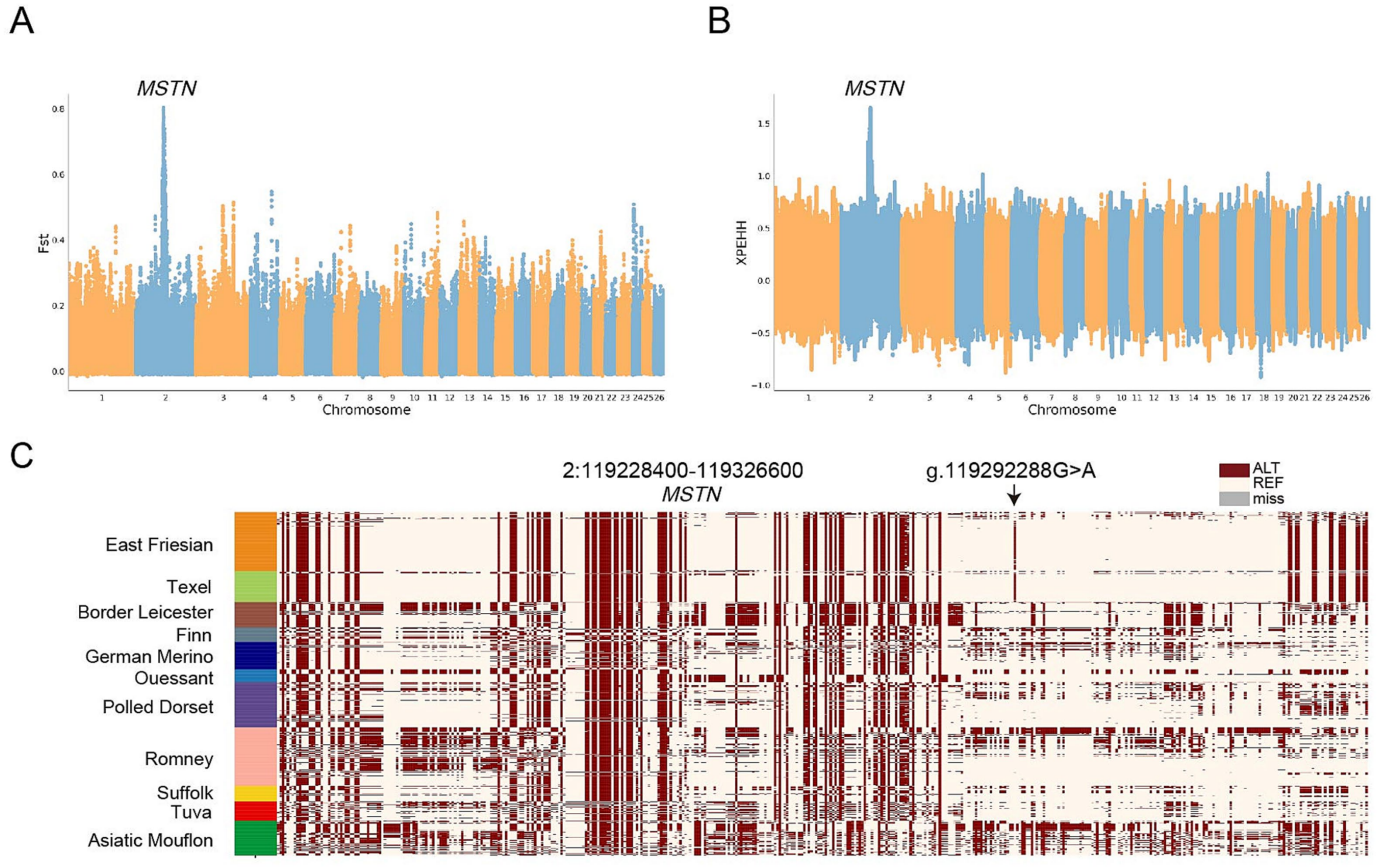
Identification of genes associated with Texel and East Friesian sheep **(A)** *F*_ST_ between Texel sheep, East Friesian sheep, and other European meat sheep breeds; **(B)** XPEHH between Texel sheep, East Friesian sheep, and other European meat sheep breeds; **(C)** SNPs with MAF > 0.05 are used to construct haplotype patterns (Chr2:119.2–119.3 Mb). The *MSTN* g.119292288G > A mutation (black arrow) was also located within the selected region.

A high frequency of the *MSTN* g.119292288G > A mutation was observed in both Texel and East Friesian sheep, suggesting a potential shared selection signature. To explore this, we conducted *F*_ST_ and XPEHH analyses between Texel sheep, East Friesian sheep, and other European meat breeds (Polled Dorset, Suffolk, and Finn sheep) (Figures 3A,B). Both methods identified the strongest selection signals in a region on chromosome 2 spanning 119.2–119.3 Mb, which encompasses the *MSTN* g.119292288G > A mutation (Supplementary Table 3). Notably, the *MSTN* locus ranked within the top 1% of genome-wide XPEHH signals, indicating exceptionally strong recent selection compared to the genomic background. Haplotype analysis revealed that Texel and East Friesian sheep presented a distinct haplotype under selection, spanning approximately 98 kb, compared with other European breeds (Figure 3C).

### 3.4 Tracing the origin of the *MSTN* g.119292288G>A mutation

**Figure 4:**
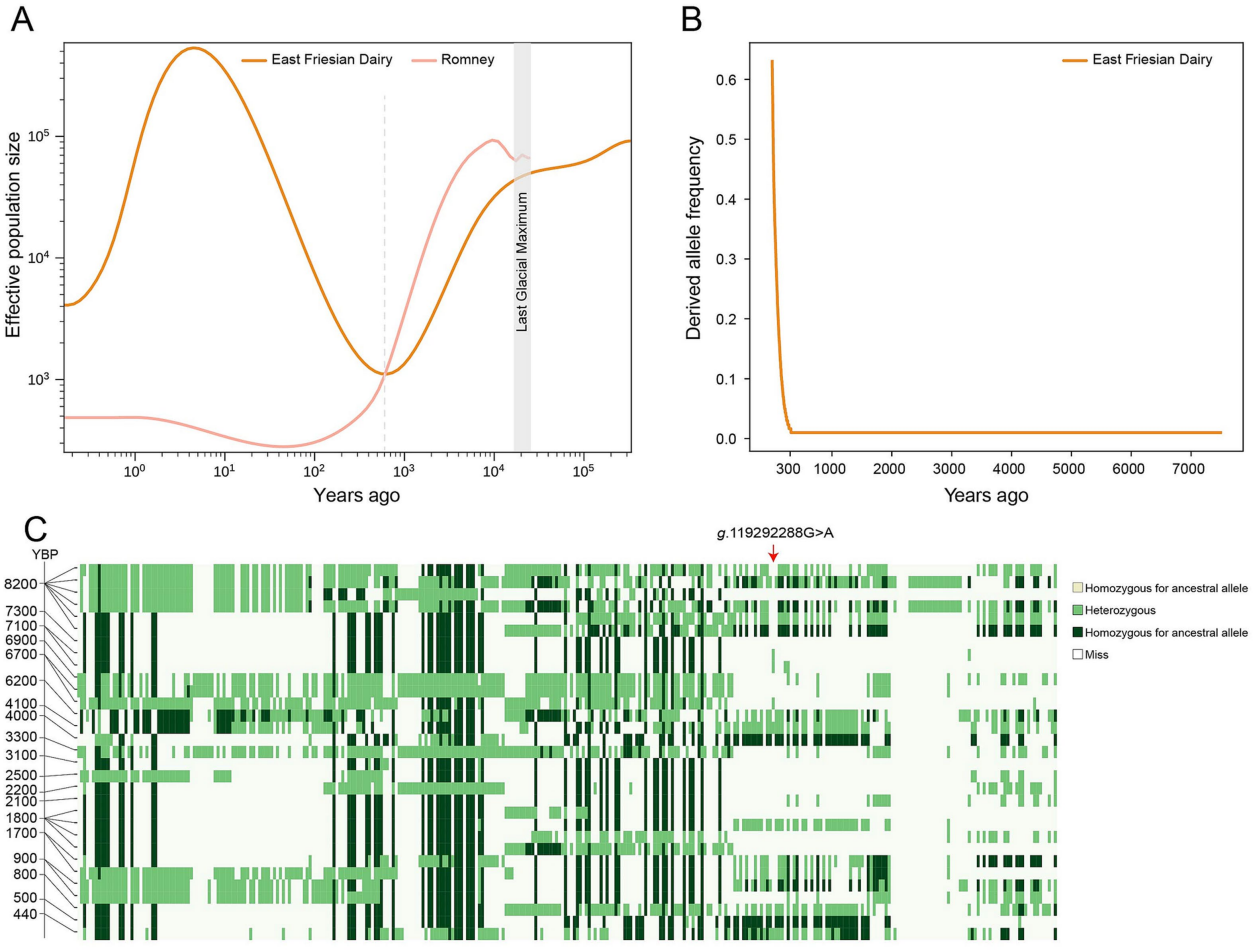
Tracing the origin and frequency trajectories of the *MSTN* g.119292288G > A mutation **(A)** Estimated divergence time between East Friesian and Romney sheep; **(B)** Allele frequency trajectory of the derived A allele in East Friesian sheep; **(C)** Haplotype patterns in the 31 ancient European sheep. The position of the mutation is indicated by a red arrow.

Due to the limited sample size of Texel sheep (*n* = 29), East Friesian sheep of 56 individuals were used for the analysis. To estimate the origin of the *MSTN* g.119292288G > A mutation, we analyzed the divergence time between East Friesian and another representative meat breed, Romney. The estimated divergence between East Friesian and Romney occurred approximately 600 years ago (Figure 4A). To further investigate the selection dynamics of the mutation, we applied ancestral recombination graph (ARG)-based analysis to trace the frequency trajectories of the mutation within East Friesian sheep. The frequency trajectory of the derived allele in East Friesian sheep showed a rapid increase in frequency over the past 300 years (Figure 4B), suggesting recent and strong positive selection. To further confirm the timing of haplotype emergence, we analyzed 31 ancient European sheep genomes dated between 8,200 and 440 years before present (YBP) (Supplementary Table 2). Within the selected region identified in modern breeds, the haplotype observed under selection was absent in ancient individuals. Moreover, the *MSTN* g.119292288G > A mutation was not found in a homozygous state in any ancient sample; most individuals were homozygous for the ancestral allele, with only sporadic heterozygous genotypes detected (Figure 4C). These findings suggested that this mutation and its associated haplotype likely arose and spread within the last 400 years.

### 3.5 Effect of the *MSTN* g.119292288G > A mutation on growth in the Hu × East Friesian hybrid population

As this mutation is primarily found in Texel and East Friesian sheep, and absent in other breeds, including Asian breeds, we studied its potential effect on muscle growth by analyzing its association with growth traits in the Hu × East Friesian hybrid population. A total of ten growth traits were recorded in this study, including body height, chest circumference, birth weight, and weaning weight and so on. Genetic association analysis revealed significant effects of g.119292288G > A locus polymorphisms on growth performance. At this biallelic locus, where G is the common allele, the heterozygous GA genotype was associated with enhanced growth performance relative to the homozygous GG genotype (Table 1). Specifically, individuals with the GA genotype presented significantly increased body weights at three developmental stages: birth weight (*p* < 0.05), weaning weight (*p* < 0.05), and 6-month weight (*p* < 0.05). In this hybrid population, individuals with the AA genotype numbered only four, rendering them unrepresentative and statistically insignificant. No statistically significant genotypic differences (*p* > 0.05) were detected in the other measured growth traits.

**Table 1:** Association analysis between *MSTN* g.119292288G > A mutation and growth traits in Hu × East Friesian hybrid population.

Growth traits	Genotypes (mean ± standard deviation)		*p*-value
	GG	GA	
Body height (cm)	52.751 ± 3.662	52.960 ± 3.677	0.734
Body length (cm)	53.103 ± 3.703	53.510 ± 3.433	0.408
Chest depth (cm)	23.768 ± 1.880	24.040 ± 1.710	0.245
Chest width (cm)	16.322 ± 1.766	16.446 ± 1.626	0.504
Chest circumference (cm)	60.334 ± 7.360	60.580 ± 7.411	0.492
Cannon circumference (cm)	7.346 ± 0.636	7.355 ± 0.572	0.758
Birth weight (kg)	3.622^A^ ± 0.816	3.763^B^ ± 0.885	0.011^*^
Weaning weight (kg)	18.196^A^ ± 3.698	18.882^B^ ± 3.744	0.005^**^
Average daily gain before weaning (kg)	0.242 ± 0.055	0.248 ± 0.059	0.126
Six-month body weight (kg)	34.778^A^ ± 7.706	36.246^B^ ± 6.674	0.036^*^

^A,B^Indicate statistically significant differences between groups (*p* < 0.05).

Asterisks denote significance levels: * represents *p* < 0.05, ** represents *p* < 0.01.

## 4 Discussion

Comprehensive sampling is essential for accurately assessing the global distribution of genetic variants. For example, the Sheep HapMap project used the Illumina OvineSNP50K Bead Chip to genotype 3,004 individuals from 71 breeds, revealing genomic regions associated with pigmentation, morphology, body size, and reproduction (37). Currently, whole-genome resequencing has emerged as a cost-effective and high-resolution approach to uncover fine-scale genomic variation and detect selection signatures (38). The integration of global-scale NGS datasets enables researchers to trace the evolutionary origins of mutations and identify breed-specific adaptations with high precision. By integrating genome-wide population data with ARG-based temporal inference, this study provides a refined reconstruction of the geographic origin, selection dynamics, and phenotypic impact of the *MSTN* g.119292288G > A mutation. Compared with previous studies that primarily described allele frequency patterns or functional effects within individual breeds, our approach enables a more comprehensive understanding of its evolutionary trajectory across global sheep populations.

By analyzing the frequency distribution of different sheep breeds worldwide revealed that *MSTN* mutations are primarily distributed in Europe and Australia. In the populations of Texel, East Friesian, and Dairy Meade sheep, allele A is the dominant allele. Dairy Meade sheep are a dairy breed developed from New Zealand’s local Coopworth sheep as the maternal line and East Friesian sheep as the paternal line (39). Therefore, Dairy Meade sheep exhibit a high frequency of the mutation, which is likely inherited from the East Friesian parent breed. Other populations with individuals carrying this mutation mostly originate from hybrid groups, such as Coopworth sheep and Australia White sheep, with a composite breed with partial Texel ancestry (40). Both Texel and East Friesian sheep originated in the North Netherlands (41), specifically from Texel Island and Friesland Province. Among all breeds globally, these two have the highest mutation frequency (>0.76). It is highly likely that this mutation originated from the northern Netherlands and subsequently emerged in hybrid breeds carrying a high frequency of this mutation through artificial selection. This mutation likely developed due to the unique climate of these northwestern Dutch islands, which is also where double-muscular cattle originated (42). The frequency of *MSTN* mutations has rapidly increased over the past 300 years. It’s quite likely that people carried out artificial breeding on East Friesian sheep in order to select those with better meat quality. This period of targeted selection coincides with the 16th–19th century western European agricultural revolution, when the cultivation of leguminous forages (e.g., clover) essential for intensive sheep farming expanded dramatically to meet the rising demand for high-quality livestock feed (43).

Notably, the mutation was absent in all currently available ancient genomes analyzed in this study. However, limited read depth and post-mortem DNA damage may increase the probability of false negatives at specific loci. In addition, sporadic heterozygous genotype calls observed in a small number of ancient individuals may be influenced by sequencing errors or damage-induced misincorporations, which are common challenges in ancient DNA analyses, particularly under low coverage conditions. Such isolated heterozygous signals, lacking consistent support across multiple reads or individuals, should therefore be interpreted cautiously. Nevertheless, even considering these technical limitations, the consistent absence of reliable derived allele signals across geographically and temporally diverse ancient samples suggests that the mutation was either extremely rare or had not yet emerged during the sampled periods. Therefore, the ancient DNA evidence remains broadly consistent with our temporal inference indicating a relatively recent origin of the *MSTN* mutation.

Internationally, research on *MSTN* gene mutations and their associations with animal growth traits has become a hot topic in the field of livestock genetic improvement. Early studies primarily focused on livestock, such as cattle (9) and sheep (8), revealing that abnormalities in muscle development caused by mutations in the *MSTN* gene play important regulatory roles in growth traits such as weight and muscle quality. Research on the *MSTN* gene in sheep has mainly focused on single breeds, Existing studies indicate that genetic variations in the *MSTN* gene are associated with traits such as sheep weight and muscle development. In this study, the significant association detected in the Hu × East Friesian hybrid population suggests that the growth-promoting effect of the *MSTN* g.119292288G > A mutation is not confined to its original breed background, but remains evident under genetic admixture, indicating a stable functional effect across genomic contexts. The superior performance of the heterozygous GA genotype supports a partial dosage effect, whereby reduced *MSTN* activity enhances muscle accretion without the potential trade-offs of complete loss of function. Due to the very small number of AA homozygotes in the hybrid population, we were unable to reliably evaluate allelic dosage effects or formally test additive versus recessive inheritance models. The superior performance of GA individuals relative to GG suggests a possible dominant or partially dominant effect of the derived allele; however, this inference should be interpreted cautiously pending validation in larger populations. The phenotypic effects of the *MSTN* mutation estimated in this study are specific to male individuals, and future studies should include both sexes to comprehensively evaluate the effects across genders. In this hybrid population, the significant but moderate effect on body weight suggests that this variant acts as a valuable quantitative modifier for growth performance. From a breeding perspective, even a 3–4% improvement in early growth traits is biologically meaningful and economically relevant for sheep production systems, as it can contribute to enhanced growth efficiency, heavier live weight at marketing, and higher economic returns when accumulated over generations and across large populations.

Tracing the origin and timing of mutations provides valuable insights into early breeding practices, which in turn can inform and assist future breeding endeavors. To enhance the precision of mutation tracing, it is advisable to consider increasing the number of studied varieties, such as East Friesian sheep. Moreover, the collection of ancient samples from diverse time periods and geographical locations can be utilized to correct the actual occurrence time of mutations (44). By correlating these findings with historical events of the corresponding eras, a more accurate understanding of early breeding activities can be achieved. This comprehensive approach holds great potential for advancing the fields of breeding and genetic research.

## 5 Conclusion

The *MSTN* g.119292288G > A mutation showed high frequencies in Texel, East Friesian, and Dairy Meade sheep, but was rare or absent in other breeds. The mutation may have originated from the northern Netherlands, specifically from Texel Island and the Friesland Province. A 98 kb haplotype under strong selection spanned the mutation in Texel and East Friesian sheep. Its frequency increased rapidly approximately 300 years ago, suggesting a recent origin and swift spread. This was further supported by its absence in 31 ancient European samples dated between 8,200 and 440 YBP. Association analysis in a Hu × East Friesian hybrid population revealed that the mutation was significantly associated with growth traits, indicating its potential as a valuable genetic marker for breeding. Moreover, this case provided a paradigm for identifying large-effect mutations contributing to economically important traits in livestock.

## Data Availability

Publicly available datasets were analyzed in this study. This sequencing data can be found in the European Nucleotide Archive (ENA) and NCBI Sequence Read Archive (SRA) under the following BioProject accession numbers: PRJEB59481, PRJEB61808, PRJEB69690, and PRJEB81145. In addition, the allele frequency data of the modern samples used in this study are publicly available at: http://animal.omics.pro/.
